# Endometrial Thickness as Diagnostic Triage for Endometrial Cancer Among Black Individuals

**DOI:** 10.1001/jamaoncol.2024.1891

**Published:** 2024-06-27

**Authors:** Kemi M. Doll, Mindy Pike, Julianna Alson, Patrice Williams, Erin Carey, Til Stürmer, Mollie Wood, Erica E. Marsh, Ronit Katz, Whitney R. Robinson

**Affiliations:** 1 Department of Obstetrics and Gynecology, Fred Hutchinson Cancer Center, University of Washington, Seattle; 2 Department of Obstetrics and Gynecology, School of Medicine, University of North Carolina at Chapel Hill, Chapel Hill; 3 Department of Epidemiology, School of Global Public Health, University of North Carolina at Chapel Hill, Chapel Hill; 4 Department of Obstetrics and Gynecology, School of Medicine, University of Michigan, Ann Arbor; 5 Department of Obstetrics and Gynecology, School of Medicine, Duke University, Durham, North Carolina

## Abstract

**Importance:**

Poor performance of the transvaginal ultrasonography triage strategy has been suggested as a contributor to racial disparity between Black individuals and White individuals in endometrial cancer (EC) stage at diagnosis in population-level simulation analyses.

**Objectives:**

To examine the false-negative probability using ultrasonography-measured endometrial thickness (ET) thresholds as triage for EC diagnosis among Black individuals and assess whether known risk factors of EC modify ET triage performance.

**Design, Setting, and Participants:**

This retrospective diagnostic study of merged abstracted electronic health record data and secondary administrative data (January 1, 2014, to December 31, 2020) from the Guidelines for Transvaginal Ultrasound in the Detection of Early Endometrial Cancer sample assessed Black individuals who underwent hysterectomy in a 10-hospital academic-affiliated health care system and affiliated outpatient practices. Data analysis was performed from January 31, 2023, to November 30, 2023.

**Exposure:**

Pelvic ultrasonography within 24 months before hysterectomy.

**Main Outcome and Measures:**

Ultrasonography performed before hysterectomy as well as demographic and clinical data on symptom presentation, endometrial characterization, and final EC diagnosis were abstracted. Endometrial thickness thresholds were examined for accuracy in ruling out EC diagnosis by using sensitivity, specificity, and negative predictive value. False-negative probability was defined as 1 − sensitivity. Accuracy measures were stratified by risk factors for EC and by factors hypothesized to influence ET measurement quality.

**Results:**

A total of 1494 individuals with a uterus (median [IQR] age, 46.1 [41.1-54.0] years) comprised the sample, and 210 had EC. Fibroids (1167 [78.1%]), vaginal bleeding (1067 [71.4%]), and pelvic pain (857 [57.4%]) were the most common presenting diagnoses within 30 days of ultrasonography. Applying the less than 5-mm ET threshold, there was an 11.4% probability that someone with EC would be classified as not having EC (n = 24). At the 4-mm (cumulative) threshold, the probability was 9.5%, and at 3 mm, it was 3.8%. False-negative probability at the 5-mm threshold was similar among EC risk factor groups: postmenopausal bleeding (12.4%; 95% CI, 7.8%-18.5%), body mass index greater than 40 (9.3%; 95% CI, 3.1%-20.3%); and age 50 years or older (12.8%; 95% CI, 8.4%-18.5%). False-negative probability was also similar among those with fibroids on ultrasonography (11.8%; 95% CI, 6.9%-18.4%) but higher in the setting of reported partial ET visibility (26.1%; 95% CI, 10.2%-48.4%) and pelvic pain (14.5%; 95% CI, 7.7%-23.9%).

**Conclusion and Relevance:**

These findings suggest that the transvaginal ultrasonography triage strategy is not reliable among Black adults at risk for EC. In the presence of postmenopausal bleeding, tissue sampling is strongly recommended.

## Key Points

**Question:**

What is the false-negative probability using ultrasonography-measured endometrial thickness (ET) thresholds as triage for endometrial cancer (EC) diagnosis among Black individuals, and do known risk factors of EC modify ET triage performance?

**Findings:**

In this diagnostic study of 1494 Black patients in a 10-hospital academic-affiliated health care system who all underwent hysterectomy, of whom 210 had EC, 11.4% with EC had ET below the diagnostic triage threshold for detection. Classic risk factors for EC (postmenopausal bleeding, age, and body mass index) did not result in improved performance of ET triage threshold.

**Meaning:**

These findings suggest that the transvaginal ultrasonography triage strategy is not reliable among Black adults at risk of EC; for Black patients with concerning symptoms, tissue biopsy is recommended to avoid misdiagnosis of EC.

## Teaser

This diagnostic study of Black patients assesses the false-negative probability and accuracy of ultrasonography-measured endometrial thickness thresholds as triage for endometrial cancer diagnosis.

## Introduction

Postmenopausal bleeding (PMB) occurs as a symptom of endometrial cancer (EC) in most people diagnosed with this condition.^1^ Current guidelines suggest that individuals presenting with PMB can undergo pelvic transvaginal ultrasonography (TVUS), and for those with an endometrial thickness (ET) measurement of 4 mm or greater, diagnostic endometrial tissue sampling is warranted.^2,3^ If the ET is less than 4 mm, then no further testing is required. Thus, TVUS is a triage strategy whose outcome either continues or halts the diagnostic process. Arguably, the error rate, or false negative probability, of such a strategy should be near zero. Published estimates on which guidelines are based report a negative predictive value (NPV) of 99% to 100%.^4^ In the largest series (n = 4833), there were 3131 women with ET less than 5 mm and only 11 EC cases reported (0.4%).^3^

However, insured Black patients with EC are less likely to receive endometrial biopsy after presenting with PMB^5^; they have reported undergoing ultrasonography with initially reassuring results^6^; and a previous simulation study using population estimates suggested that the TVUS triage approach may miss cases of EC among Black individuals due to their greater prevalence of fibroids and nonendometrioid histologic types.^7,8,9^ Before revising the TVUS triage approach, it is necessary to incorporate data directly from clinical care encounters of Black patients. The Guidelines for Transvaginal Ultrasound in the Detection of Early Endometrial Cancer (GUIDE-EC) project (eAppendix in Supplement 1) was funded to study EC diagnostic accuracy, health care delivery, and quality of care for Black individuals. The goals of this first analysis are to (1) examine the false-negative probability of the TVUS ET triage thresholds, (2) assess whether known risk factors of EC modify ET threshold accuracy, and (3) identify clinical risk factors, if any, of EC cases with ET below threshold.

## Methods

### Sample Selection

In this diagnostic study, we used a searchable federation of electronic health information (data warehouse) and administrative data from a large academic medical health system, with information from the 10 hospitals and hundreds of affiliated practices to create the GUIDE-EC sample. The process included capturing clinical data from administrative codes and electronic health record (EHR) abstraction as described in detail in the GUIDE-EC sample description (eAppendix in Supplement 1). Two race variables from the data warehouse were used to identify Black race; if either were selected, the record was included. Black race was then confirmed on abstraction by checking the self-reported race identification in the EHR. If there were multiple races selected that included Black, the individuals were included. For this analysis, the sample was restricted to those undergoing pelvic ultrasonography with a documented ET measurement before hysterectomy. This study was reviewed by the University of Washington Institutional Review Board Human Subjects Division and determined to have no more than minimal risk; therefore, informed consent was not required.

### Pelvic Ultrasonographic Data

Pelvic ultrasonographic data came from direct imaging reports in the native EHR, scanned reports from other health systems into the EHR, physician note summaries of pelvic ultrasonography results, and any other clinician note (eg, nursing note or telephone note) that contained pelvic ultrasonography results within 24 months of hysterectomy. Ultrasonographic information source quality was tiered by the most direct (formal ultrasonographic report in native EHR) to the most indirect (scanned physician note from outside facility reporting outside ultrasonography results). When the ultrasonographic data source was physician notes, these notes were further categorized into those including a detailed report, defined as at least 3 measurements related to ultrasonography, vs those that were classified as summary reports (<3 measurements). From ultrasonography results, uterine, endometrial, and fibroid measurements were abstracted, alongside any text-based descriptions of size and measurement of the endometrium. We did not collect data on potential adverse events due to pelvic ultrasonography.

### Variable Definitions

Endometrial thickness was abstracted directly from pelvic ultrasonographic reports and physician notes, both native to the EHR and as media files scanned from outside facilities. The measurement was captured in units as reported (millimeters or centimeters) and later transformed to a consistent unit (millimeters). In instances where units were missing, the entire ultrasonographic report underwent clinical review to determine the appropriate unit. In records where the ET was described in text but not measured (eg, “thin endometrial stripe”), the text was abstracted but the individual and their ultrasonography results were excluded from this primary analysis.

Endometrial cancer diagnosis was identified from the data warehouse administrative *Current Procedural Terminology* codes and confirmed by abstracted data from the hysterectomy pathology reports, as the reference standard for diagnosis. Discrepant records in which *Current Procedural Terminology* codes did not match abstracted pathology classification underwent additional clinical review, including information from symptoms and diagnosis free text, biopsy, and/or dilation and curettage and, if needed, direct evaluation of the EHR by a gynecologist or gynecologic oncologist.

### Statistical Analysis

Descriptive statistics, including numbers (percentages) for categorical data and medians (IQRs) for continuous variables, were used to generate summary tables and simple frequencies of demographic and clinical data. Factors hypothesized to influence abstracted ultrasonographic data quality were also summarized and stratified by both ET thresholds and EC status. We compared factors using χ^2^ and Fisher exact tests for categorical variables and 2-tailed, unpaired *t* tests for continuous variables. The ET thresholds were defined as less than 3 mm, less than 4 mm, and less than 5 mm, with the rest grouped as 5 mm or more, consistent with current and past professional society guidelines. Of note, these groups are not mutually exclusive, allowing for the possibility of a given ET measurement falling into multiple categories simultaneously. Accuracy of the ET threshold to rule out a case of EC was assessed in several ways. Sensitivity, specificity, and NPV, with 95% CIs, were calculated for each ET threshold individually. We calculated the false-negative probability (1 − sensitivity) to show the probability of a person with EC being classified as not having EC at each threshold. We then stratified by predefined factors hypothesized to affect accuracy: (1) risk factors for EC (age ≥50 years, presence of PMB, and body mass index [BMI; calculated as weight in kilograms divided by height in meters squared]), (2) endometrial visibility (partial vs full), (3) clinical history of fibroids, (4) presence of fibroids on pelvic ultrasonography, and (5) pelvic pain as a presenting diagnosis or symptom. We used descriptive statistics to examine differences in demographic and clinical factors between EC cases with ET measurements below the 5-mm threshold and EC cases with ET measurements above the 5-mm threshold. Finally, we performed sensitivity analyses of removing a small number of ultrasonograms found to have been reported within the study window but occurred on a date outside the study window and limiting to only those ultrasonograms performed within 90 days of hysterectomy. Sample selection for the GUIDE-EC sample was guided by an expected 10% prevalence of EC in a population of individuals who underwent hysterectomy. In our population of 1494 Black patients who underwent hysterectomy with ET measurement, a total of 210 had EC. With a power of 80% and α = .05, we can detect differences in sensitivity of 8 percentage points or more from a target sensitivity of 80%. Data analysis was performed from January 31, 2023, to November 30, 2023. All statistical analyses were conducted using Stata, version 18 (StataCorp LLC).^10^

## Results

Sample demographics and characteristics are detailed in Table 1, stratified by the final diagnosis of EC. From the original GUIDE-EC Sample (N = 3455), we excluded 750 who had no evidence of undergoing pelvic ultrasonography, 266 who had a pelvic ultrasonogram with nonvisible endometrium, and 945 who had pelvic ultrasonography information that did not include any endometrial information. The final sample for this analysis was 1494 individuals with a uterus (median [IQR] age, 46.1 [41.1-54.0] years) (eFigure 1 in Supplement 1). On the basis of explicit gender identity recorded in the EHR and/or gender-related indication for hysterectomy, there were 6 gender-expansive individuals (transgender men, nonbinary, or other noncisgender identity) included.

**Table 1.** Characteristics of Black Individuals Who Underwent Pelvic Ultrasonography and Hysterectomy in the GUIDE-EC Sample (2014-2020)^a^

Characteristic	Overall (N = 1494)	Endometrial cancer (n = 210)	No endometrial cancer (n = 1284)
Age at hysterectomy, median (IQR), y	46.1 (41.1-54.0)	64.4 (57.1-70.6)	45.1 (40.3-50.1)
Transgender identity and/or gender dysphoric diagnosis			
Yes	6 (0.4)	0	6 (0.5)
No	1488 (99.6)	210 (100)	1278 (99.5)
BMI, median (IQR)	34.4 (29.4-40.3)	36.0 (31.5-42.8)	34.2 (29.2-40.0)
BMI group			
≤40	820 (73.3)	108 (66.7)	712 (74.4)
>40	299 (26.7)	54 (33.3)	245 (25.6)
Presenting symptoms and diagnoses (within 30 d of presenting event)			
Postmenopausal bleeding	275 (18.4)	161 (76.7)	114 (8.9)
Fibroids	1167 (78.1)	137 (65.2)	1030 (80.2)
Any bleeding	1067 (71.4)	108 (51.4)	959 (74.7)
Pelvic or abdominal pain	857 (57.4)	83 (39.5)	774 (60.3)
Enlarged uterus	655 (43.8)	69 (32.9)	586 (45.6)
Anemia	638 (42.7)	65 (31.0)	573 (44.6)
Pelvic mass	653 (43.7)	61 (29.1)	592 (46.1)
Fatigue or lightheadedness	459 (30.7)	52 (24.8)	407 (31.7)
Abnormal Papanicolaou test result	343 (23.0)	52 (24.8)	291 (22.7)
Urinary symptoms	329 (22.0)	37 (17.6)	292 (22.7)
Menopausal symptoms	266 (17.8)	106 (50.5)	160 (12.5)
Endometrial hyperplasia	112 (7.5)	52 (24.8)	60 (4.7)
Clinical history (within 2 y of hysterectomy)			
Smoking			
Never	994 (66.5)	139 (66.2)	855 (66.6)
Past	248 (16.6)	44 (21.0)	204 (15.9)
Current	239 (16.0)	26 (12.4)	213 (16.6)
Transfusion history	160 (10.7)	20 (9.5)	140 (10.9)
Family history of cancer			
Breast	370 (24.8)	42 (20.0)	328 (25.6)
Ovarian	99 (6.6)	12 (5.7)	87 (6.8)
Uterine	63 (4.2)	9 (4.3)	54 (4.2)
Cervical	35 (2.3)	5 (2.4)	30 (2.3)
Mental health diagnoses			
Depression	225 (15.1)	20 (9.5)	205 (16.0)
Bipolar	33 (2.2)	<5 (<2.4)^b^	>29 (>2.3)^b^
Anxiety	211 (14.1)	30 (14.3)	181 (14.1)
PTSD	15 (1.0)	<5 (<2.4)^b^	>11 (>0.9)^b^
Charlson Comorbidity Index			
0	1008 (67.5)	101 (48.1)	907 (70.7)
1	289 (19.4)	55 (26.2)	234 (18.2)
≥2	196 (13.1)	54 (25.7)	142 (11.1)
Insurance type at hysterectomy			
Private	912 (61.0)	73 (34.8)	839 (65.3)
Medicare	248 (16.6)	113 (53.8)	135 (10.5)
Medicaid	183 (12.3)	10 (4.8)	173 (13.5)
Agency^c^	<5 (<0.3)^b^	<5 (<2.4)^b^	<5 (<0.4)^b^
Tricare	33 (2.2)	<5 (<2.4)^b^	>29 (>2.3)^b^
Self-pay	117 (7.8)	12 (5.7)	105 (8.2)
Endometrial thickness, median (IQR), mm	8 (5-12.7)	15.8 (9-25)	7.3 (4.8-11)
Hysterectomy facility			
Academic primary hospital	424 (28.4)	159 (75.7)	265 (20.6)
Academic community hospital	1070 (71.6)	51 (24.3)	1019 (79.4)

Abbreviations: BMI, body mass index (calculated as weight in kilograms divided by height in meters squared); GUIDE-EC, Guidelines for Transvaginal Ultrasound in the Detection of Early Endometrial Cancer; PTSD, posttraumatic stress disorder.

^a^ Results are reported as No. (%) unless otherwise noted.

^b^ Data for cells with fewer than 5 participants are suppressed per institutional review board approval requirements.

^c^ Agency includes skilled nursing facilities, home health agencies, Veteran Affairs, and other governmental programs.

### Pelvic Ultrasonographic Quality and Data Reporting

Detailed information on pelvic ultrasonographic data quality is reported in Table 2. Ultrasonograms for this analysis were limited to the first pelvic ultrasonography received that included an ET measurement (1494 scans). There were 26 ultrasonograms whose data were reported within the abstraction window but occurred more than 24 months from hysterectomy. With regard to abstracted data quality, most ultrasonography was performed at academic-affiliated community facilities, and nearly all (1461 [97.8%]) had result information accessible via native ultrasonographic reports (645 [43.2%]), scanned media including ultrasonographic reports and physician notes from outside facilities (546 [36.6%]), or physician notes native to the EHR reporting data from documented ultrasonography (270 [18.1%]). Within all physician note sources, including native and scanned, most ultrasonographic information reported (284 [89.3%]) was detailed. Regarding the type of pelvic ultrasonography, most included a transvaginal approach (1149 [76.9%]), few were abdominal only (94 [6.3%]), and in 251 (16.8%) this information was explicitly missing. Most records (1226 [82.1%]) had timely contextual clinical notes from within 30 days before or after ultrasonography. Regarding potential factors influencing ET measurement, 1114 ultrasonograms (74.6%) reported the presence of fibroids, including 212 (14.2%) submucosal. Endometrial thickness visibility was fully visible in 1258 (84.2%) and partially visible in 236 (15.8%).

**Table 2.** Pelvic Ultrasonographic Data and Quality by Endometrial Thickness in the 1494 Participants

Characteristic	No. (%) of participants by endometrial thickness, mm				Total No. (%) of participants (N = 1494)	*P* value^a^
	<3 (n = 116)	<4 (n = 228)	<5 (n = 355)	≥5 (n = 1139)		
Ultrasonography location						
Academic hospital	21 (18.1)	40 (17.5)	63 (17.8)	169 (14.8)	232 (15.5)	.17
Academic community hospital	54 (46.6)	92 (40.4)	118 (33.2)	352 (30.9)	470 (31.5)	
Academic community practitioner	<5 (<4.3)^b^	<5 (<2.2)^b^	10 (2.8)	28 (2.5)	38 (2.5)	
Private hospital	5 (4.3)	21 (9.2)	40 (11.3)	133 (11.7)	173 (11.6)	
Private practitioner	23 (19.8)	48 (21.1)	82 (23.1)	327 (28.7)	409 (27.4)	
Community hospital	<5 (<4.3)^b^	<5 (<2.2)^b^	<5 (<1.4)^b^	>25 (>2.2)^b^	29 (1.9)	
Unknown	10 (8.6)	23 (10.1)	39 (11.0)	104 (9.1)	143 (9.6)	
Clinical notes within 30 d						
Yes, notes are accessible	96 (82.8)	185 (81.1)	284 (80.0)	942 (82.7)	1226 (82.1)	.09
Yes, notes not accessible	<5 (<4.3)^b^	8 (3.5)	14 (3.9)	36 (3.2)	50 (3.4)	
No notes identified	7 (6.0)	18 (7.9)	28 (7.9)	100 (8.8)	128 (8.6)	
Other	<5 (<4.3)^b^	<5 (<2.2)^b^	6 (1.7)	5 (0.4)	11 (0.7)	
Missing	8 (6.9)	15 (6.6)	23 (6.5)	56 (4.9)	79 (5.3)	
Ultrasonographic information source						
Ultrasonographic report	66 (56.9)	120 (52.6)	169 (47.6)	476 (41.8)	645 (43.2)	.19
Physician notes	16 (13.8)	39 (17.1)	67 (18.9)	203 (17.8)	270 (18.1)	
Scanned media^c^	33 (28.5)	66 (29.0)	114 (32.1)	432 (37.9)	546 (36.6)	
Ultrasonographic report	28 (84.9)	58 (89.2)	101 (89.4)	393 (91.6)	494 (91.1)	
Physician notes	5 (15.2)	7 (10.8)	12 (10.6)	36 (8.4)	48 (8.9)	
Other	<5 (<4.3)^b^	<5 (<2.2)^b^	5 (1.4)	28 (2.5)	33 (2.2)	
Physicians note information quality (scanned and native)						
Detailed report	19 (90.5)	42 (91.3)	70 (88.6)	214 (89.5)	284 (89.3)	.82
Summary	<5 (<4.3)^b^	<5 (<2.2)^b^	9 (11.4)	25 (10.5)	34 (10.7)	
Approach of pelvic ultrasonography						
Transvaginal and abdominal	41 (35.3)	91 (39.9)	141 (39.7)	527 (46.3)	668 (44.7)	.11
Transvaginal only	44 (37.9)	79 (34.7)	127 (35.8)	354 (31.1)	481 (32.2)	
Abdominal only	5 (4.3)	12 (5.3)	20 (5.6)	74 (6.5)	94 (6.3)	
Not reported	26 (22.4)	46 (20.2)	67 (18.9)	184 (16.2)	251 (16.8)	
Uterine measurement						
Measurement present	112 (96.6)	217 (95.2)	334 (94.1)	1082 (95.0)	1416 (94.8)	.62
Descriptive words	<5 (<4.3)^b^	<5 (<2.2)^b^	5 (1.4)	18 (1.6)	23 (1.5)	
Absent	<5 (<4.3)^b^	9 (4.0)	16 (4.5)	39 (3.4)	55 (3.7)	
Fibroids present on ultrasonogram	91 (78.5)	179 (78.5)	272 (76.6)	842 (73.9)	1114 (74.6)	.31
Submucosal	10 (8.6)	23 (10.1)	35 (9.9)	177 (15.5)	212 (14.2)	.003
Endometrial thickness visibility report						
Visible	101 (87.1)	193 (84.7)	293 (82.5)	965 (84.7)	1258 (84.2)	.32
Partially visible	15 (12.9)	35 (15.4)	62 (17.5)	174 (15.3)	236 (15.8)	
Endometrial cancer	8 (6.9)	20 (8.8)	24 (6.8)	186 (16.3)	210 (14.1)	<.001

^a^ *P* values denote comparison of all endometrial thickness thresholds.

^b^ Data for cells with fewer than 5 participants are suppressed per institutional review board approval requirements. Each threshold is inclusive to evaluate clinical use of 3-, 4-, or 5-mm triage cutoff points.

^c^ Scanned media indicated documents scanned into the electronic health record from outside hospitals or facilities.

There were no significant differences by the ET thickness threshold across any of these quality indicators (Table 2). There were slightly more native ultrasonographic reports among ultrasonograms with less than 3-mm, less than 4-mm, and less than 5-mm ET measurements compared with greater than 5 mm. Among scanned media, physician notes were slightly more common than scanned ultrasonographic reports in the less than 3-mm group. Overall, physician notes had an equivalent balance of detailed vs summary reports across the ET thresholds. Fibroid presence was also similarly distributed. When stratified by presence or absence of EC, there were no significant differences across these quality indicators (eTable 1 in Supplement 1). Overall, 24 of 355 people (6.8%) with ET less than 5 mm on ultrasonograms had EC.

### ET Threshold Performance

Table 3 reports the numbers of EC cases by ET thresholds. In each 3-, 4-, and 5-mm threshold, there are EC cases among individuals below the threshold. Using 3 mm as the ET threshold, 8 EC cases would have been misclassified as not having EC (false-negative probability of 3.8%; 95% CI, 1.7%-7.4%). At the 4-mm (cumulative) threshold, that false-negative probability is 9.5% (95% CI, 5.9%-14.3%), and at the 5-mm threshold, it is 11.4% (95% CI, 7.5%-16.5%). Considering TVUS triage strategy overall, the sensitivity decreases with increasing measurement threshold with 96.2% (95% CI, 92.6%-98.3%) sensitivity of the 3-mm threshold and 88.6% (95% CI, 83.5%-92.5%) sensitivity with the 5-mm threshold. In sensitivity analysis, there were 4 cases of EC among the 26 individuals with pelvic ultrasonographic information from greater than 24 months before hysterectomy. When removing these scans, results varied by less than 0.2 percentage point (eTable 2 in Supplement 1). When further restricting to only to ultrasonograms performed within 90 days of hysterectomy, there were slight decreases in false-negative probability to 2.8% (95% CI, 0.8%-7.0%) at 3 mm, 7.7% (95% CI, 3.9%-13.3%) at 4 mm, and 8.4% (95% CI, 4.4%-14.2%) at 5 mm (eTable 3 in Supplement 1). The NPV ranged from 54% to 95% based on threshold and stratified risk factors (below). Because NPV is highly dependent on sample size and the balance between EC and non-EC cases, its comparison across study populations is limited (eTable 4 in Supplement 1).

**Table 3.** Sensitivity, Specificity, and False-Negative Probability of Endometrial Cancer by Endometrial Thickness Thresholds in the 1494 Participants

Endometrial thickness threshold, mm	No. of patients		% (95% CI)				
	Endometrial cancer (n = 210)	No endometrial cancer (n = 1284)	Sensitivity		Specificity		False-negative probability
**Threshold: 3 mm**							
<3	8	108	96.2 (92.6-98.3)	8.4 (7.0-10.1)		3.8 (1.7-7.4)	
≥3	202	1176					
**Threshold: 4 mm**							
<4	20	208	90.5 (85.7-94.1)	16.2 (14.2-18.3)		9.5 (5.9-14.3)	
≥4	190	1076					
**Threshold: 5 mm**							
<5	24	331	88.6 (83.5-92.5)	25.8 (23.4-28.3)		11.4 (7.5-16.5)	
≥5	186	953					

### Stratification by Risk Factors for EC

For those younger than 50 years, there were only 23 total EC cases and no cases with ET less than 5 mm. Among those 50 years or older, there were 187 total EC cases, including 24 with ET less than 5 mm (false-negative probability of 12.8%; 95% CI, 8.4%-18.5%). In stratifying by age, therefore, the false-negative probability was similar to the overall group (Figure; eTable 5 in Supplement 1). Among the 275 people with PMB as part of their presentation, the false-negative probability was also similar, ranging from 4.3% (95% CI, 1.8%-8.8%) at the 3-mm threshold to 12.4% (95% CI, 7.8%-18.5%) at the 5-mm threshold.

**Figure.**
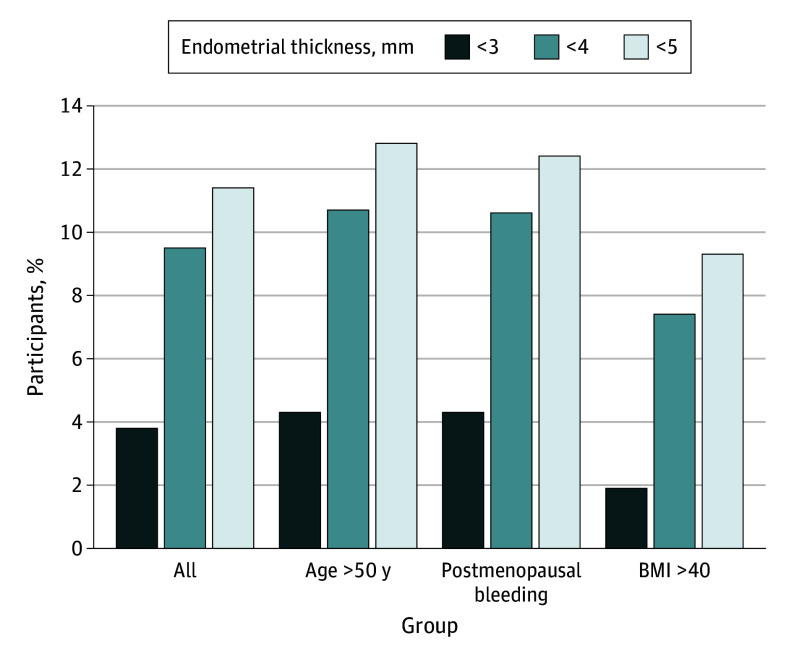
False-Negative Probability of Endometrial Thickness Measurement for Endometrial Cancer Diagnostic Triage Among Black Individuals BMI indicates body mass index (calculated as weight in kilograms divided by height in meters squared).

When stratifying among individuals with BMI information (n = 1119 [74.9%]), there were 108 cases of EC at a BMI of 40 or less and 54 cases of EC at a BMI greater than 40. Among those with a BMI greater than 40, the false-negative probability improved slightly, with a range of 1.9% (95% CI, 0.0%-9.9%) at the 3-mm threshold to 9.3% (95% CI, 3.1%-20.3%) at the 5-mm threshold. These results presented with the 100% scale can be found in eFigure 2 in Supplement 1.

### Stratification by Factors Hypothesized to Affect ET Measurement Accuracy

With regard to the potential influence of ET visibility on measurement accuracy, there were 23 cases of EC among patients with ET reported to be partially visible on ultrasonograms compared with 187 EC cases among those with full ET visibility (eTable 6 in Supplement 1). Among the partially visible group with EC, the false-negative probability ranged from 13.0% (95% CI, 2.8%-33.6%) at 3 mm to 26.1% (95% CI, 10.2%-48.4%) at 5 mm compared with 2.7% (95% CI, 0.9%-6.1%) (3 mm) to 9.6% (95% CI, 5.8%-14.8%) (5 mm) for those with fully visible ET. Among those with a history of fibroids, the false-negative probability ranged from 4.4% (95% CI, 1.6%-9.3%) (3 mm) to 13.1% (95% CI, 8.0%-20.0%) (5 mm), and the group with no history of fibroids was small (n = 79) overall, resulting in few cases of EC. Among those with fibroids detected on pelvic ultrasonography in which the ET was measured, the false-negative probability ranged from 4.4% (95% CI, 1.6%-9.4%) (3 mm) to 11.8% (95% CI, 6.9%-18.4%) (5 mm) compared with 2.7% (95% CI, 0.3%-9.4%) (3 mm) to 10.8% (95% CI, 4.8%-20.2%) (5 mm) for those without fibroids present. Overall, 899 of 1114 people (80.7%) with ultrasonography-detected fibroids had a fully visible endometrium compared with 359 of 380 people (94.5%) without fibroids. Among the 857 individuals with pelvic pain, there were 83 cases of EC. In these cases, the false-negative probability was slightly higher, ranging from 4.8% (95% CI, 1.3%-11.9%) at 3 mm to 14.5% (95% CI, 7.7%-23.9%) at 5 mm.

### Characteristics of Those With EC Above and Below the 5-mm Threshold

Overall, 24 EC cases (11.4%) had an ET measurement of less than 5 mm. Table 4 details clinical characteristics by those with EC and measured ET above and below the 5-mm ET threshold. Notable differences (>10%) include a higher prevalence of fibroids, pelvic pain, and enlarged uterus among the less than 5-mm group. This group also had a longer interval between ultrasonography and hysterectomy (median, 91 days; IQR, 53-162) compared with the 5-mm or greater group (median, 55 days; IQR, 37-106 days). Among those below the ET threshold, endometrial hyperplasia, Charlson Comorbidity Index of 0, and biopsy before hysterectomy were less common. There were no statistically significant differences for any factors, aside from age at hysterectomy.

**Table 4.** Characteristics of Participants With Endometrial Cancer Below or Above the 5-mm Endometrial Thickness Threshold^a^

Characteristic	Total participants with endometrial cancer (n = 210)	Endometrial thickness <5 mm (n = 24)	Endometrial thickness ≥5 mm (n = 186)	*P* value
Age at hysterectomy, median (IQR), y	64.4 (57.1-70.6)	66.6 (61.3-73.3)	64.0 (56.4-70.2)	.02
BMI, median (IQR)	36.0 (31.5-42.8)	36.8 (32.1-40.8)	35.9 (31.4-42.8)	.99
BMI group				
≤40	108 (66.7)	14 (73.7)	94 (65.7)	.61
>40	54 (33.3)	5 (26.3)	49 (34.3)	
Postmenopausal bleeding	161 (76.7)	20 (83.3)	141 (75.8)	.76
Fibroids	137 (65.2)	18 (75.0)	119 (64.0)	.52
Any bleeding	108 (51.4)	11 (45.8)	97 (52.2)	.40
Pelvic or abdominal pain	83 (39.5)	12 (50.0)	71 (38.2)	.53
Enlarged uterus	69 (32.9)	10 (41.7)	59 (31.7)	.63
Anemia	65 (31.0)	6 (25.0)	59 (31.7)	.58
Pelvic mass	61 (29.1)	9 (37.5)	52 (28.0)	.44
Fatigue or lightheadedness	52 (24.8)	8 (33.3)	44 (23.7)	.23
Abnormal Papanicolaou test result	52 (24.8)	5 (20.8)	47 (25.3)	.67
Urinary symptoms	37 (17.6)	<5 (<20.8)^b^	>33 (>17.7)^b^	.29
Menopausal symptoms	106 (50.5)	14 (58.3)	92 (49.5)	.57
Endometrial hyperplasia	52 (24.8)	<5 (20.8)^b^	>45 (>24.2)^b^	.07
Smoking				
Never	139 (66.2)	15 (62.5)	124 (66.7)	.07
Past	44 (20.9)	7 (29.2)	37 (19.9)	
Current	26 (12.4)	<5 (<20.8)^b^	>24 (>12.9)^b^	
Transfusion history	20 (9.5)	<5 (<20.8)^b^	>18 (>9.7)^b^	.14
Family history of cancer				
Breast	42 (20.0)	7 (29.2)	35 (18.8)	.28
Ovarian	12 (5.7)	<5 (<20.8)^b^	>8 (>4.3)^b^	.37
Uterine	9 (4.3)	<5 (<20.8)^b^	>5 (2.7)^b^	.60
Cervical	5 (2.4)	<5 (<20.8)^b^	>1 (>0.5)^b^	1.0
Mental health diagnosis				
Depression	20 (9.5)	<5 (<20.8)^b^	>13 (>7.0)^b^	.71
Bipolar disorder	<5 (<2.4)^b^	<5 (<20.8)^b^	<5 (<2.7)^b^	1.0
Anxiety	30 (14.3)	<5 (12.5)^b^	>23 (>12.4)^b^	1.0
PTSD	<5 (<2.4)^b^	<5 (<20.8)^b^	<5 (<2.7)^b^	1.0
Charlson Comorbidity Index, mean (SD)	1.4 (2.2)	1.3 (1.7)	1.4 (2.3)	
0	101 (48.1)	9 (37.5)	92 (49.5)	.48
1	55 (26.2)	8 (33.3)	47 (25.3)	
≥2	54 (25.7)	7 (29.2)	47 (25.3)	
Presence of fibroids on ultrasonogram	136 (64.8)	16 (66.7)	130 (64.5)	.84
Submucosal	17 (8.1)	<5 (20.8)^b^	>12 (>6.5)^b^	1.0
Time between ultrasonography and hysterectomy, median (IQR), d	60 (39-113)	91 (53-162)	55 (37-106)	.17

Abbreviations: BMI, body mass index (calculated as weight in kilograms divided by height in meters squared); PTSD, posttraumatic stress disorder.

^a^ Data are presented as number (percentage) of participants unless otherwise indicated.

^b^ Data for cells with fewer than 5 participants are suppressed per institutional review board approval requirements.

## Discussion

The GUIDE-EC sample was created to analyze quality of care in EC diagnosis among Black individuals. There were 24 cases of EC below the 5-mm threshold that would trigger an endometrial biopsy, resulting overall in 11.4% of EC cases with the potential for misclassification. This is a concerning error rate for a triage strategy that would terminate further workup and provide false reassurance to both patients and physicians. This result contributes to an increasing body of work questioning the wisdom of the TVUS triage strategy.^8,9^ It may be the case that the TVUS triage for endometrial biopsy is no longer a preferred strategy in the setting of increasing EC rates for all.^11,12^

In this analysis, we directly assessed factors that may influence TVUS accuracy suggested by prior work.^6,7^ We found that false-negative probability increased when the ET visibility was partial. Improved visualization may have led to different ET measurements that would have captured the underlying EC risk. Consequently, although ET could be a reliable indicator of biopsy need, its accuracy remains suboptimal among too many people. Fibroids were also associated with lower ET visibility; however, the difference in false-negative probability when stratified by fibroids alone was small, suggesting other mediators of this association. Individuals experiencing pelvic pain had higher false-negative probabilities—a potential sign that increased discomfort with the vaginal ultrasonography probe might lead to a shorter, lower-quality study as technicians seek to minimize harm. The data capture of histologic type was too incomplete to assess this factor.

### Limitations

This study has some limitations. We chose not to include those with ultrasonographic reports that omitted the ET measurement or reported a nonvisible ET in this analysis. Although clinically these could be interpreted as thin endometrium that do not require biopsy, we chose a more conservative approach and only assessed those with numerical ET measurements. In this manner, we may be underestimating the frequency of potential failure of the ultrasonography triage strategy given individual clinician decision-making in the setting of a nonvisible or absent ET measurement. This population is also defined by hysterectomy to allow for definitive final diagnostic accuracy. We likely captured a more symptomatic noncancer group compared with symptomatic Black women and gender-expansive people with benign uterine disease who do not undergo hysterectomy.^13,14,15^ If symptomatic Black individuals with benign disease who do not undergo hysterectomy are more likely to have thinner ET compared with those who do (our sample), then the risk of EC cases below threshold (eg, percentage with EC among those with ET<5 mm) would be overestimated. For this reason, we report that statistic but emphasize the false-negative probability (1 − sensitivity) that would not be subject to this bias. Finally, this sample omits EC cases that are not treated with hysterectomy, which may occur in young people with grade 1 endometrial cancer, those medically incapable of undergoing surgery, and those with disease so advanced that surgery is no longer an option. In the last case, we may be omitting the very cases resulting from erroneous ultrasonography triage that cause delayed diagnoses. We acknowledge that race may act as a rough proxy for an undefined biological variable that may drive the ultrasonographic results and accuracy for Black patients. We do not have ancestry analysis in this study.

Use of TVUS to assess need for endometrial biopsy among Black individuals at risk for EC is not a reliable triage strategy. In the presence of concerning symptoms, we recommend tissue sampling. It is important to consider that no strategy is failproof. For example, a meta-analysis^16^ on endometrial biopsy alone reported that although biopsy had a 99.6% EC detection rate among postmenopausal patients, inadequate sampling rates ranged from 0% to 54% across 39 studies. Inadequate sampling should prompt further evaluation with hysteroscopy and/or dilation and curettage in the setting of continued bleeding. Ongoing work^17^ should continue to innovate beyond tissue biopsy in the diagnostic evaluation of EC to develop less invasive methods that do not sacrifice accuracy.

## Conclusions

In this diagnostic study, among Black patients who underwent hysterectomy, a significant proportion of those with EC had ET below the range of diagnostic thresholds for detection. Classic risk factors for EC (postmenopausal bleeding, age, and body mass index) did not result in improved performance of the ET triage thresholds. These findings suggest that the transvaginal ultrasonography triage strategy is not reliable among Black adults at risk for EC. In the presence of postmenopausal bleeding, tissue sampling is strongly recommended.

## Notes

Supplement 1. **eAppendix.** GUIDE EC Sample: Summary of Data Collection, Abstraction, and Quality Assessments **eFigure 1.** Flowchart of inclusion and exclusion of patient cases from the GUIDE-EC full sample **eTable 1.** Pelvic Ultrasound Data and Quality by Endometrial Cancer Diagnosis (n = 1494) **eTable 2.** Sensitivity, specificity, and classification of endometrial cancer by endometrial thickness thresholds in participants with US within 24-month time period (n = 1122) **eTable 3.** Sensitivity, specificity, and false negative probability of endometrial cancer by endometrial thickness thresholds in those with US≤90 days before hysterectomy (n = 612) **eTable 4.** Endometrial thickness by Endometrial Cancer Status, stratified by risk factors for EC **eFigure 2.** False Negative Probability by ET threshold, overall and stratified by risk factors of EC **eTable 5.** Endometrial thickness by Endometrial Cancer Status, stratified by risk factors for EC **eTable 6.** Endometrial thickness by Endometrial Cancer Status, stratified by factors hypothesized to influence ET measurement quality (n = 1494)

Supplement 2.
Data Sharing Statement
