# Supplementary material for: Endometrial Thickness as Diagnostic Triage for Endometrial Cancer Among Black Individuals
Source: JAMA Oncol. 2024 Jun 27;10(8):1068–76. doi: 10.1001/jamaoncol.2024.1891 (PMC11211989; doi:10.1001/jamaoncol.2024.1891)
Supplement: Supplement 1. — eAppendix. GUIDE EC Sample: Summary of Data Collection, Abstraction, and Quality Assessments eFigure 1. Flowchart of inclusion and exclusion of patient cases from the GUIDE-EC full sample eTable 1. Pelvic Ultrasound Data and Quality by Endometrial Cancer Diagnosis (n = 1494) eTable 2. Sensitivity, specificity, and classification of endometrial cancer by endometrial thickness thresholds in participants with US within 24-month time period (n = 1122) eTable 3. Sensitivity, specificity, and false negative probability of endometrial cancer by endometrial thickness thresholds in those with US≤90 days before hysterectomy (n = 612) eTable 4. Endometrial thickness by Endometrial Cancer Status, stratified by risk factors for EC eFigure 2. False Negative Probability by ET threshold, overall and stratified by risk factors of EC eTable 5. Endometrial thickness by Endometrial Cancer Status, stratified by risk factors for EC eTable 6. Endometrial thickness by Endometrial Cancer Status, stratified by factors hypothesized to influence ET measurement quality (n = 1494) [file jamaoncol-e241891-s001.pdf]

Supplementary Online Content

Doll KM, Pike M, Alson J, et al. Endometrial thickness as diagnostic triage for endometrial cancer in Black people. *JAMA Oncol.* Published online June 27, 2024. doi:10.1001/jamaoncol.2024.1891

eAppendix. GUIDE EC Sample: Summary of Data Collection, Abstraction, and Quality Assessments

eFigure 1. Flowchart of inclusion and exclusion of patient cases from the GUIDE-EC full sample.

eTable 1. Pelvic Ultrasound Data and Quality by Endometrial Cancer Diagnosis (n=1,494)

eTable 2. Sensitivity, specificity, and classification of endometrial cancer by endometrial thickness thresholds in participants with US within 24-month time period (n=1,122)

eTable 3. Sensitivity, specificity, and false negative probability of endometrial cancer by endometrial thickness thresholds in those with US ≤90 days before hysterectomy (n=612)

eTable 4. Endometrial thickness by Endometrial Cancer Status, stratified by risk factors for EC

eFigure 2. False Negative Probability by ET threshold, overall and stratified by risk factors of EC

eTable 5. Endometrial thickness by Endometrial Cancer Status, stratified by risk factors for EC

eTable 6. Endometrial thickness by Endometrial Cancer Status, stratified by factors hypothesized to influence ET measurement quality (n=1,494)

This supplementary material has been provided by the authors to give readers additional information about their work.

© 2025 Doll KM et al. *JAMA Oncology*.

eAppendix. GUIDE-EC Sample: Summary of data collection, abstraction, and quality control.

Data Sources. To create a sample of symptomatic Black individuals undergoing hysterectomy, we employed a searchable federation of electronic health information and administrative data from a large academic medical health system, which included data from 10 hospitals and hundreds of affiliated practices. We queried the database for structured clinical data and supplemented this with free text and imaging reports from the Electronic Health Record (EHR), all captured by a team of professional abstractors. This multi-step process corresponds with methods described in a prior study.[1]

Sample Selection. Those eligible for the study included state residents over age 18 who self-identified as Black or African American and who underwent hysterectomy between April 4, 2014 and December 31, 2020. Administrative billing codes were used to identify all hysterectomies performed during the eligible date range, including an International Classification of Diseases (ICD)-9 to ICD10 crosswalk to ensure full capture (Table 1). People were excluded if they were pregnant at the time of surgery (as defined by hysterectomy with birth-related ICD-9 and 10 codes), had prior or active breast cancer diagnoses, cancers where vaginal bleeding would be non-uterine in origin (vulva, vagina), or placental-site cancers at the time of hysterectomy. Records were excluded if the hysterectomy occurred within three months of the EPIC go-live date at the facility where the hysterectomy occurred, to ensure ability to fully capture symptoms and diagnosed elements leading up to surgical intervention.

Capture of Structured Data. After identifying individuals eligible for the sample, we additionally captured, from the health system's data warehouse, date of birth, confirmed race, age, height and weight, and insurance type at the time of hysterectomy. For clinical information, we captured date of surgery, diagnosis of all malignant neoplasms of the pelvis at the time of hysterectomy, and specified physician-and hospital-billed diagnostic and Common Procedural Terminology (CPT) codes (including pelvic transvaginal ultrasound (TVUS), endometrial biopsy (EMB), dilation and curettage (D&C) and gynecologic symptoms and diagnoses) within 24 months prior to hysterectomy. We captured all variables required for the Charlson Comorbidity Index (CCI), a validated index to classify prognostic comorbidity, at the time of hysterectomy.[2]

Capture of Unstructured Data.
We developed an EHR data abstraction tool using REDCap, along with a corresponding written protocol. This tool was designed to capture information about symptoms, diagnoses, imaging and procedure results, and comorbid conditions for the included patients. Before finalizing the EHR abstraction tool and protocol, we sampled 18 observed patient records from the data warehouse and conducted a pilot abstraction. Following this, we refined the order of abstracted variables and deepened protocol-level detail on how to obtain pelvic ultrasound (US) measurement information from different sources. The priority for each record was identification of the first pelvic ultrasonography performed in the 24 months prior to hysterectomy, to serve as a proxy for the index symptom date. The abstractors then reviewed a 30-day window before and after this date to capture presenting symptoms and active diagnoses at the time of the diagnostic process. If no pelvic ultrasonography or other imaging (magnetic resonance imaging - MRI) was performed or available, abstractors were instructed to use the first biopsy or D&C as a proxy for the index symptom date. In charts where no imaging or biopsy were performed, the date of hysterectomy was designated as a proxy for the index symptom date, with symptoms abstracted from the preceding 30 days. (Data from these latter two scenarios are not included in this current analysis, as these patients did not have ultrasonography prior to hysterectomy.) We purposefully planned overlap with several variables captured in the structured dataset to capture possible events and services completed outside of the health system but present elsewhere in the medical record. The time frame of sample identification and data abstraction is illustrated in the Figure.

Demographic data abstracted included date of birth, age at hysterectomy, procedure facility, insurance category, and race confirmation. Abstracted clinical data of presenting symptoms and diagnoses from physician and nursing notes in the index diagnostic process window. Abstracted data on mental health diagnosis, smoking status, and family history of cancer were abstracted from the entire study period prior to hysterectomy. Indications for diagnostic procedures, hysterectomies, and post-operative diagnoses from pathology reports were also captured. Symptoms were characterized as present, reported absent, or absent from the record (no documentation for presence or absence). From ultrasonography results, uterine, endometrial, and fibroid measurements were abstracted, alongside identification of performing facility.

© 2025 Doll KM et al. *JAMA Oncology*.

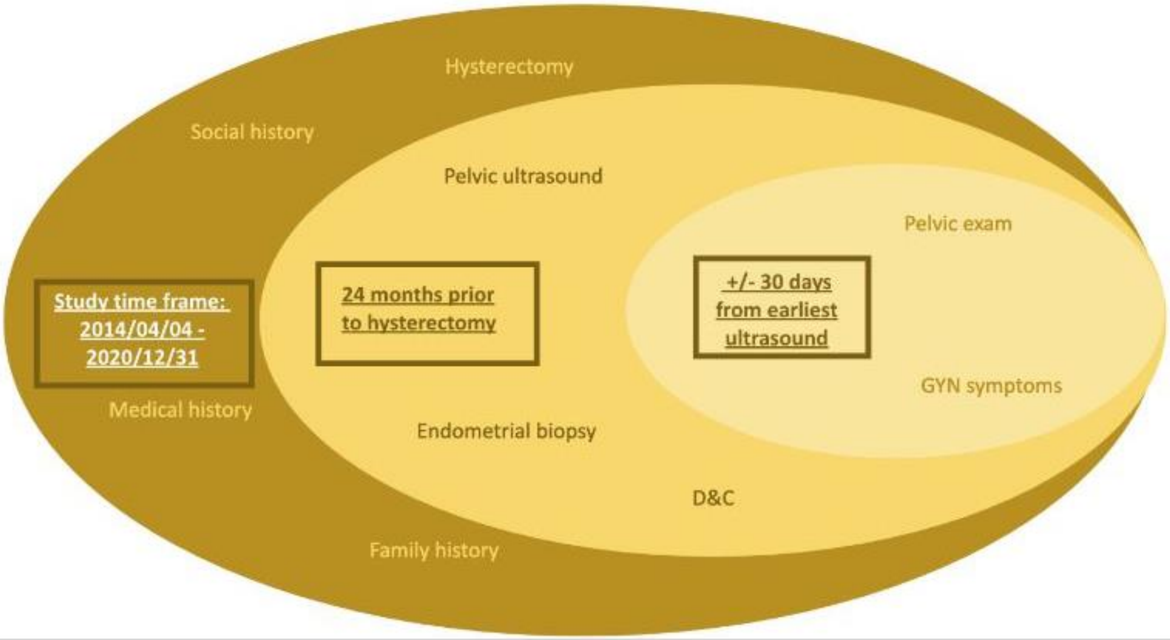

This illustrates the time windows employed to create the GUIDE-EC Sample. Records from 2014 to 2020 were captured for all hysterectomy, by CPT codes. After application of exclusion criteria, family history, medical history, and social history were abstracted. Pelvic ultrasound, endometrial biopsy, and D&C procedures were obtained from up to 24 months prior to hysterectomy. The first such event, prioritized by pelvic ultrasound, then any tissue biopsy procedures, was identified, then detailed abstraction of presentation symptoms and diagnoses were captured in the 30 days prior and following the event for full clinical context.

Abstraction Quality Control. During the abstraction, we adhered to the protocol guide and kept a corollary log of questions from abstractors requiring scientific or clinical consult; together, these served as active documents with auditable updates based on abstractors' feedback and ongoing quality assurance review. A team of four abstractors with over 20 years of cumulative experience completed all data abstraction. They could initiate secondary review for any data ambiguity, and 5% of all records from each site were chosen at random, via a Stata coding command, in 2-week intervals to undergo double abstraction.[3] Double abstracted records were compared to identify discrepancies (numeric and text based). If any were discovered, a manual review was initiated to organize flags into typographic errors vs. differences in abstractor's interpretations. The latter were resolved through group discussion in consultation with clinical leads (K.D., E.C.) followed by appropriate protocol updates. All data was abstracted into REDCap and merged with administrative data for the final analytic dataset. A cleaning process was then undertaken to reconcile inconsistent procedure dates and eliminate any empty records. The dataset was de-identified.

Data Cleaning. After the merging and de-identification of the dataset, from January 2023 to May 2023, data cleaning included assessment of missingness, collapsing variables as needed, and reconciling free text entries from the abstraction process. Each line of free text for all abstracted records was reviewed and either recategorized into appropriate existing variables or a new variable was created, as appropriate. The reconciliation process included making additions to the symptom and diagnostic variables, recategorization of ultrasonography location into de-identified categories (e.g., Private practice OBGYN), identifying rare EC histology types (e.g., Endometrial stromal sarcoma) and reviewing extreme numerical values of key variables (e.g., BMI, ultrasonography measurements) for clinical feasibility and possible exclusion. Abstracted free-text data were used to re-apply exclusion criteria for cancer and pregnancy that initial administrative code definitions missed. Following these steps, a total of 14 remaining discrepant records

© 2025 Doll KM et al. *JAMA Oncology*.

underwent a final clinical re-review to resolve data discrepancies between administrative data and EHR-based abstraction data.

1. Robinson WR, Mathias JG, Wood ME, et al. Ethnoracial Differences in Premenopausal Hysterectomy: The Role of Symptom Severity. *Obstetrics and gynecology*. 2023;142(2):350-359. doi:10.1097/AOG.0000000000005225
2. Charlson ME, Pompei P, Ales KL, MacKenzie CR. A new method of classifying prognostic comorbidity in longitudinal studies: Development and validation. *J Chronic Dis*. 1987;40(5):373-383. doi:10.1016/0021-9681(87)90171-8
3. 19. StataCorp. . Published online 2023.

© 2025 Doll KM et al. *JAMA Oncology*.

eFigure 1. Flowchart of inclusion and exclusion of patient cases from the GUIDE-EC full sample.

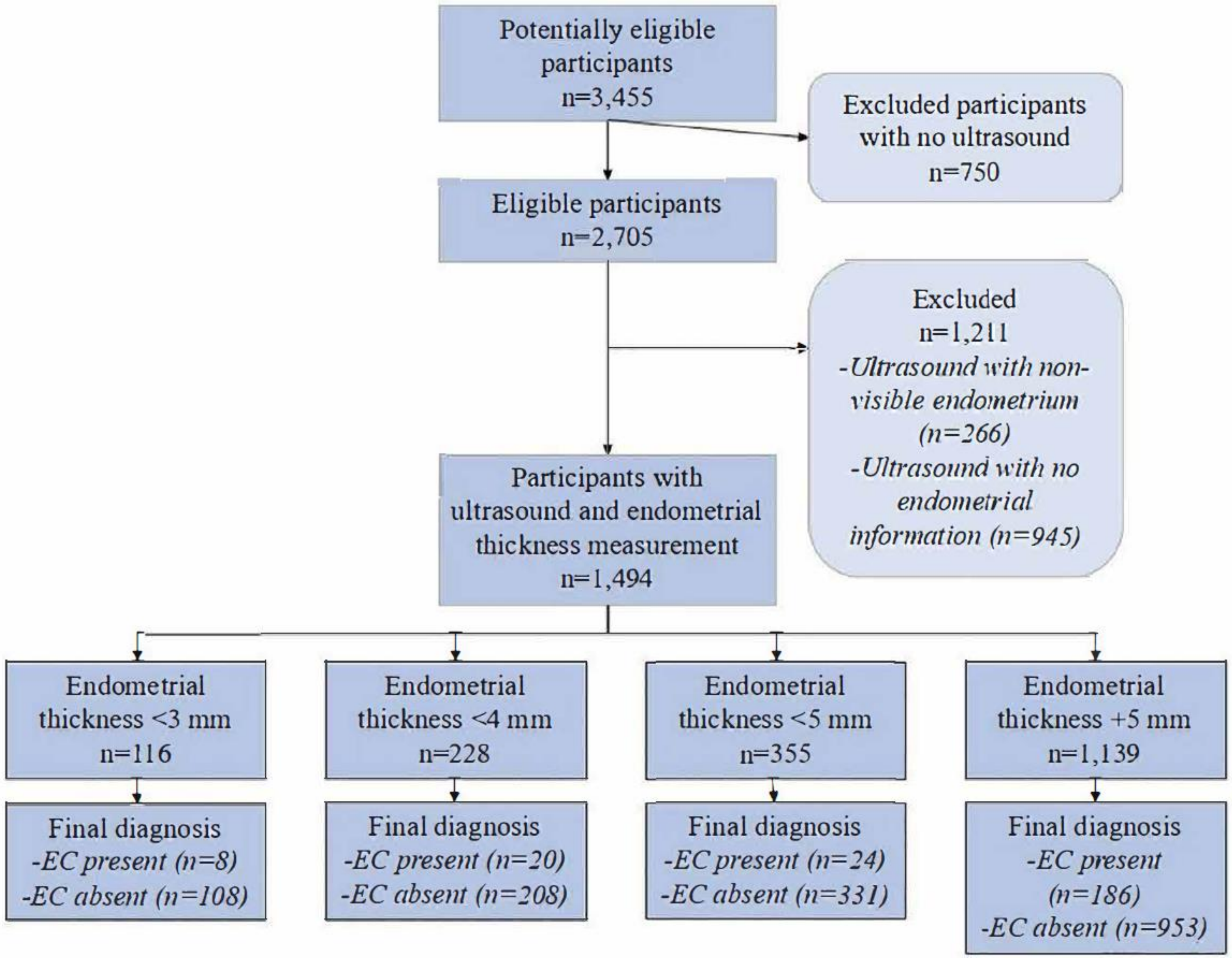

© 2025 Doll KM et al. *JAMA Oncology*.

eTable 1. Pelvic Ultrasonography Data and Quality by Endometrial Cancer Diagnosis (n=1,494)

US Data and Quality Characteristic	No Endometrial Cancer (n=1,284)	Endometrial Cancer (n=210)
US location		
Academic hospital	219 (17.1)	13 (6.2)
Academic community hospital	416 (32.4)	54 (25.7)
Academic community provider	32 (2.5)	6 (2.9)
Private hospital	132 (10.3)	41 (19.5)
Private provider	337 (26.3)	72 (34.4)
Community hospital	26 (2.0)	<5 (<2.4)[a]
Unknown	122 (9.5)	>20 (>9.5)[a]
Clinical notes within 30 days		
Yes, notes are accessible	1,033 (80.5)	193 (91.9)
Yes, notes not accessible	43 (3.4)	7 (3.3)
No notes identified	121 (9.4)	7 (3.3)
Other	10 (0.8)	<5 (<2.4)[a]
Missing	77 (6.0)	<5 (<2.4)[a]
US Information Source		
Ultrasound report	601 (46.8)	44 (21.0)
Physician notes	227 (17.7)	43 (20.5)
Scanned media	430 (33.5)	116 (55.2)
Ultrasound report	388 (91.1)	106 (91.4)
Physician notes	38 (8.9)	10 (8.6)
No information available	8 (0.6)	<5 (<2.4)
Other	18 (1.4)	<5 (<2.4)
Physicians note information quality (scanned + native)		
Detailed report	239 (90.2)	45 (84.9)
Summary	26 (9.8)	8 (15.1)
Approach of Pelvic US		
Transvaginal and abdominal	576 (44.9)	92 (43.8)
Transvaginal only	420 (32.7)	61 (29.1)
Abdominal only	85 (6.6)	9 (4.3)
Not reported	203 (15.8)	48 (22.9)
Uterine measurement		
Measurement present	1,223 (95.3)	>190 (>90.5)[a]
Descriptive words	19 (1.5)	<5 (<2.4)[a]
Absent	42 (3.3)	13 (6.2)
Fibroids present on US	978 (76.2)	136 (64.8)
Submucosal	195 (34.5)	17 (30.9)

© 2024 American Medical Association. All rights reserved.

eTable 1. Pelvic Ultrasonography Data and Quality by Endometrial Cancer Diagnosis (n=1,494) continued

US Data and Quality Characteristic	No Endometrial Cancer (n=1,284)	Endometrial Cancer (n=210)
ET visibility report		
Visible	1,071 (83.4)	187 (89.1)
Partially visible	213 (16.6)	23 (11.0)

Notes: Values listed as N (%)
a Values for cells with <5 participants are suppressed per IRB approval requirements

© 2024 American Medical Association. All rights reserved.

eTable 2. Sensitivity, specificity, and classification of endometrial cancer by endometrial thickness thresholds in participants with US within 24-month time period (n=1,122)

	EC	No EC	Sensitivity (95% CI)	Specificity (95% CI)	False negative probability (95% CI)
**Threshold: 3 mm**					
<3 mm	8	106	96.1% (92.5-98.3)	8.4% (6.9-10.1)	3.9% (1.7-7.5)
≥3 mm	198	1,156			
**Threshold: 4 mm**					
<4 mm	19	206	90.8% (86.0-94.4)	16.3% (14.3-18.5)	9.2% (5.6-14.0)
≥4 mm	187	1,056			
**Threshold: 5 mm**					
<5 mm	23	323	88.8% (83.7-92.8)	25.6% (23.2-28.1)	11.2% (7.2-16.3)
≥5 mm	183	939			

© 2024 American Medical Association. All rights reserved.

eTable 3. Sensitivity, specificity, and false negative probability of endometrial cancer by endometrial thickness thresholds in those with US ≤90 days before hysterectomy (n=612)

	EC	No EC	Sensitivity (95% CI)	Specificity (95% CI)	False negative probability (95% CI)
Total	143	469			
**Threshold: 3 mm**					
<3 mm	<5	>40	97.2% (93.0-99.2)	8.7% (6.4-11.7)	2.8% (0.8-7.0)
≥3 mm	139	428			
**Threshold: 4 mm**					
<4 mm	11	83	92.3% (86.7-96.1)	17.7% (14.3-21.5)	7.7% (3.9-13.3)
≥4 mm	132	386			
**Threshold: 5 mm**					
<5 mm	12	116	91.6% (85.8-95.6)	24.7% (20.9-28.9)	8.4% (4.4-14.2)
≥5 mm	131	353			

© 2024 American Medical Association. All rights reserved.

eTable 4. Endometrial thickness by Endometrial Cancer Status, stratified by risk factors for EC

Endometrial Thickness	Total	EC	No EC	NPV (95% CI)
**Total**				
	N=1494	N=210	N=1284	
<3 mm	116	8	108	93.1% (86.9-97.0)
<4 mm	228	20	208	91.2% (86.8-94.6)
<5 mm	355	24	331	93.2% (90.1-95.6)
5+ mm	1139	186	953	NA[b]
**Age ≥50 years**				
	N=518	N=187	N=331	
<3 mm	47	8	39	83.0% (69.2-92.4)
<4 mm	94	20	74	78.7% (69.1-86.5)
<5 mm	129	24	105	81.4% (73.6-87.7)
5+ mm	389	163	226	NA[b]
**Post-menopausal bleeding**				
	N=275	N=161	N=114	
<3 mm	15	7	8	53.3% (26.6-78.7)
<4 mm	36	17	19	52.8% (35.5-69.6)
<5 mm	45	20	25	55.6% (40.0-70.4)
5+ mm	230	141	89	NA[b]
**BMI>40**				
	N=299	N=54	N=245	
<3 mm	14	<5[a]	>12[a]	92.9% (66.1-99.8)
<4 mm	37	<5[a]	>32[a]	89.2% (74.6-97.0)
<5 mm	62	5	57	91.9% (82.2-97.3)
5+ mm	237	49	188	NA[b]

a Values for cells with <5 participants are suppressed per IRB approval requirements
b Not applicable

© 2024 American Medical Association. All rights reserved.

eFigure 2. False Negative Probability of ET Measurement for EC Diagnostic Triage Among Black Individuals

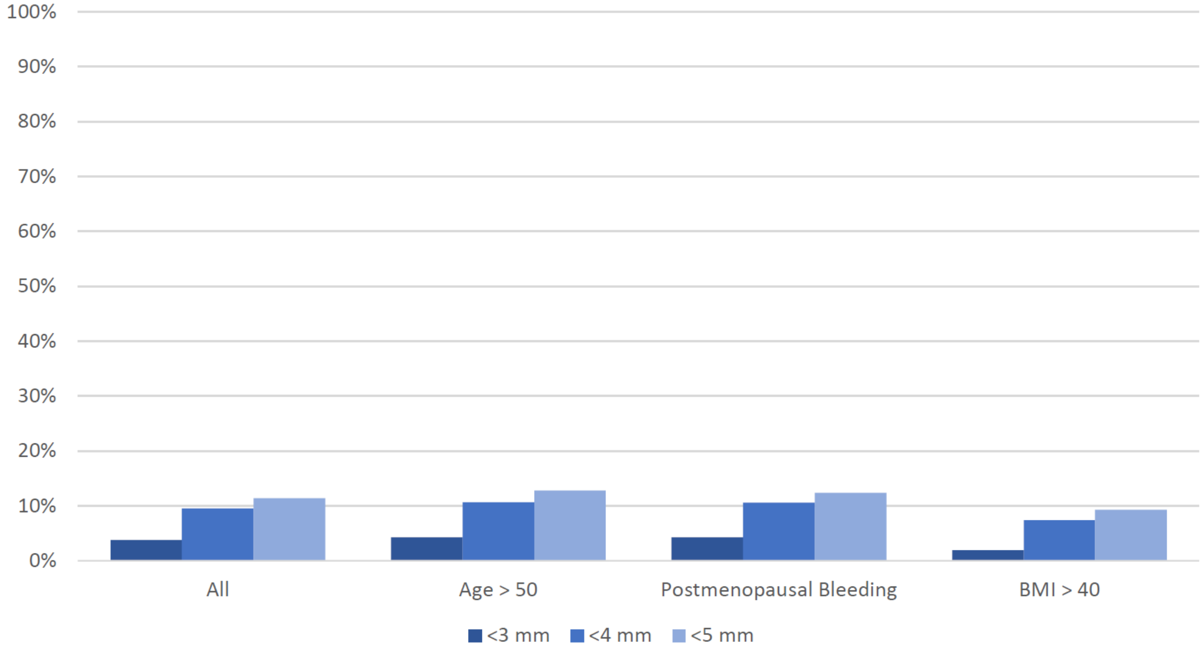

© 2024 American Medical Association. All rights reserved.

eTable 5. Endometrial thickness by Endometrial Cancer Status, stratified by risk factors for EC

Endometrial Thickness	Total	EC	No EC	**False negative probability** (95% CI)
**Age ≥50 years**				
	N=518	N=187	N=331	
<3 mm	47	8	39	4.3% (1.9-8.3)
<4 mm	94	20	74	10.7% (6.7-16.0)
<5 mm	129	24	105	12.8% (8.4-18.5)
5+ mm	389	163	226	NA[b]
**Post-menopausal bleeding**				
	N=275	N=161	N=114	
<3 mm	15	7	8	4.3% (1.8-8.8)
<4 mm	36	17	19	10.6% (6.3-16.4)
<5 mm	45	20	25	12.4% (7.8-18.5)
5+ mm	230	141	89	NA[b]
**BMI>40**				
	N=299	N=54	N=245	
<3 mm	14	<5[a]	>12[a]	1.9% (0.0-9.9)
<4 mm	37	<5[a]	>32[a]	7.4% (2.1-17.9)
<5 mm	62	5	57	9.3% (3.1-20.3)
5+ mm	237	49	188	NA[b]

a Values for cells with <5 participants are suppressed per IRB approval requirements
b Not applicable

© 2024 American Medical Association. All rights reserved.

eTable 6. Endometrial thickness by Endometrial Cancer Status, stratified by factors hypothesized to influence ET measurement quality (n=1,494)

Endometrial Thickness	Total	Endometrial Cancer	No Endometrial Cancer	**False negative probability** (95% CI)
**Visible**				
<3 mm	101	5	96	2.7% (0.9-6.1)
<4 mm	93	14	179	7.5% (4.2-12.2)
<5 mm	293	18	275	9.6% (5.8-14.8)
5+ mm	965	169	796	NA[b]
**Partially Visible**				
<3 mm	15	<5[a]	>8[a]	13% (2.8-33.6)
<4 mm	35	6	29	26.1% (10.2-48.4)
<5 mm	62	6	56	26.1% (10.2-48.4)
5+ mm	174	17	157	NA[b]
**No history of fibroids ^**				
<3 mm	6	<5[a]	>2[a]	5.3% (0.1-26.0)
<4 mm	10	<5[a]	>5[a]	10.5% (1.3-33.1)
<5 mm	14	<5[a]	>10[a]	10.5% (1.3-33.1)
5+ mm	65	17	48	NA[b]
**History of fibroids**				
<3 mm	92	6	86	4.4% (1.6-9.3)
<4 mm	182	14	168	10.2% (5.7-16.6)
<5 mm	282	18	264	13.1% (8.0-20.0)
5+ mm	885	119	766	NA[b]
**No presence of fibroids**				
<3 mm	25	<5[a]	>20[a]	2.7% (0.3-9.4)
<4 mm	49	6	43	8.1% (3.0-16.8)
<5 mm	83	8	75	10.8% (4.8-20.2)
5+ mm	297	66	231	NA[b]
**Presence of fibroids**				
<3 mm	91	6	85	4.4% (1.6-9.4)
<4 mm	179	14	165	10.3% (5.7-16.7)
<5 mm	272	16	256	11.8% (6.9-18.4)
5+ mm	842	120	722	NA[b]
**No pelvic pain**[c]				
<3 mm	22	<5[a]	>15[a]	3.4% (0.7-9.6)
<4 mm	44	6	38	6.8% (2.5-14.3)
<5 mm	68	9	59	10.2% (4.8-18.5)
5+ mm	255	79	176	NA[b]

© 2024 American Medical Association. All rights reserved.

eTable 6. Endometrial thickness by Endometrial Cancer Status, stratified by factors hypothesized to influence ET measurement quality (n=1,494) continued

	**Total**	**Endometrial Cancer**	**No Endometrial Cancer**	**False negative probability** (95% CI)
**Endometrial Thickness**				
**Pelvic Pain**				
<3 mm	65	<5[a]	>60[a]	4.8% (1.3-11.9)
<4 mm	138	11	127	13.3% (6.8-22.5)
<5 mm	219	12	207	14.5% (7.7-23.9)
5+ mm	638	71	567	NA[b]

a Values for cells with <5 participants are suppressed per IRB approval requirements
b Not applicable
c Limited to those with documented absence of symptoms

© 2024 American Medical Association. All rights reserved.
