# Supplementary material for: Endometrial Thickness as Diagnostic Triage for Endometrial Cancer Among Black Individuals
Source: JAMA Oncol. 2024 Jun 27;10(8):1068–76. doi: 10.1001/jamaoncol.2024.1891 (PMC11211989; doi:10.1001/jamaoncol.2024.1891)
Supplement: Supplement 2. — Data Sharing Statement [file jamaoncol-e241891-s002.pdf]

Data Sharing Statement

Doll. Endometrial Thickness as Diagnostic Triage for Endometrial Cancer Among Black Individuals. *JAMA Oncol*. Published June 27, 2024. doi:10.1001/jamaoncol.2024.1891

Data
Data available: No
